# Non coding RNA analysis in fibrolamellar hepatocellular carcinoma

**DOI:** 10.18632/oncotarget.23325

**Published:** 2017-12-15

**Authors:** Benjamin A. Farber, Gadi Lalazar, Elana P. Simon, William J. Hammond, David Requena, Umesh K. Bhanot, Michael P. La Quaglia, Sanford M. Simon

**Affiliations:** ^1^ Laboratory of Cellular Biophysics, The Rockefeller University, New York, 10065 NY, USA; ^2^ Division of Pediatric Surgery, Department of Surgery, Memorial Sloan-Kettering Cancer Center, New York, 10065 NY, USA; ^3^ Pathology Core Facility Memorial Sloan-Kettering Cancer Center, New York, 10065 NY, USA

**Keywords:** pediatric cancer, fusion protein, microRNA, lncRNAs, liver cancer

## Abstract

Fibrolamellar hepatocellular carcinoma (FLC) is a rare primary liver cancer found in adolescents and young adults without underlying liver disease. A deletion of ~400 kD has been found in one copy of chromosome 19 in the tumor tissue of all patients tested. This produces a fusion of the genes DNAJB1 and PRKACA which, in turn, produces a chimeric transcript and protein. Transcriptomic analysis of the tumor has shown upregulation of various oncologically relevant pathways, including EGF/ErbB, Aurora Kinase A, pak21 and wnt. To explore other factors that may contribute to oncogenesis, we examined the microRNA (miRNA) and long non-coding RNA (lncRNA) expression in FLC. The non-coding RNA expression profile in tumor tissue samples is distinctly different from the adjacent normal liver and from other liver tumors. Furthermore, miRZip knock down or over expression of certain miRNAs led to changes in the levels of coding genes that recapitulated changes observed in FLC, suggesting mechanistically that the changes in the cellular levels of miRNA are not merely correlative. Thus, in addition to serving as diagnostic tools for FLC, non-coding RNAs may serve as therapeutic targets.

## INTRODUCTION

Fibrolamellar hepatocellular carcinoma (FLC) receives its name from the distinct intratumoral lamellar bands of collagen that are visualized interspersed between large polygonal cells with vesiculated nuclei and large nucleoli on H&E images [1, 2]. While classified as a variant of conventional hepatocellular carcinoma (HCC), FLC differs in a number of important ways. FLC is typically found in adolescents and young adults without underlying liver disease, such as viral hepatitis or cirrhosis [2–4], and elevations in serum alpha fetoprotein (AFP) are not typically seen. Additionally, FLC has fewer chromosomal changes, less genomic heterogeneity and fewer mutations compared to HCC [5, 6]. Finally, the changes in the transcriptome and proteome in the FLC tumor are distinct from those in HCC [7].

The first key to the pathogenesis of FLC was the identification of a deletion of ~400 kD in one copy of chromosome 19 in the tumor tissue but not in the adjacent normal liver tissue [8]. The deletion was found in all tumor samples in the absence of any other significant recurrent changes in the genomic DNA [9], but not in surrounding paired normal liver parenchyma. The deletion results in a fusion gene of the first exon of a heat shock protein, *DNAJB1*, with the second through tenth exon of the catalytic subunit of protein kinase A, *PRKACA*. A chimeric RNA transcript and protein were found in the tumor tissue of every patient tested. This chimeric fusion protein retains the full enzymatic kinase activity [8]. The DNAJB1-PRKACA chimera has since been validated in several studies and may be considered as a defining mutation in this cancer [7, 8, 10, 11]. While the rest of the genomic DNA of FLC is relatively unremarkable, the transcriptome reveals many upregulated oncologically relevant pathways, including epidermal growth factor ((EGF), v-Erb-B2 Avian Erythroblastic Leukemia Viral Oncogene (ErbB), Aurora Kinase A and Wingless type integration (*wnt*) site family of signaling proteins [7]. These transcriptomic signatures are remarkable both in the magnitude of some of the changes as well as the consistency from patient-to-patient.

Non-coding RNAs, both microRNA (miRNA) and long-noncoding RNA (lncRNA) have been implicated to play major roles in biology ranging from embryology to cancer development. It has been estimated that up to 60% of the human genome may be under miRNA regulation [12–14]. Increasingly, the dysregulation of miRNA has been demonstrated in various cancers, and can function similarly to oncogenes and tumor suppressor genes [15]. Review of miRNA differential expression across a broad spectrum of tumors and their respective normal tissues have shown varied miRNA profiles with the ability to interact at multiple steps in a diverse set of cellular pathways [15, 16]. Additionally, miRNA have been implicated as distinct regulators of metastases [17].

LncRNAs are typically > 200 nucleotides and are not believed to be translated into proteins. Many lncRNAs are conserved through evolution, suggesting they may have a functional role. They have been shown to affect the transcription of specific genes, regulate mRNA processing, control alternative splicing, regulate protein translation, and be involved in epigenetic modification of DNA. LncRNAs have already been implicated in cancers such as leukemia, colorectal carcinoma, and in liver cancer [18–20]. Over the past ten years changes in specific lncRNAs have been shown to not only correlate with progression and outcome [21] but have been demonstrated to affect PTEN expression [22] as well as implied to play a role in metastasis [23].

To gain further understanding of the tumor biology, we investigated non-coding RNA in FLC. An examination of the lncRNA in FLC, in contrast to the adjacent normal tissue, showed distinct increases in specific transcripts. The pattern of changes was consistent across patients and distinct from changes previously reported in hepatocellular carcinoma or cholangiocarcinoma. Some lncRNA, which where found to be significantly increased in other liver cancers, such as HULC (highly upregulated in liver cancer), HOTAIR and HOTTIP only showed a mild increase in FLC; while others, such as H19, showed a decrease. An examination of miRNA in FLC tissue, in contrast to the adjacent normal tissue was conducted and demonstrated 176 mature miRNA that were changed significantly in their expression levels, |log2 fold change| > 1, FDR <0.01. A number of these miRNA have been previously implicated, from other systems, in the regulation of the level of coding changes in specific target genes. For example, miR-548p was decreased in FLC and a known target, the mRNA for FZD10, is significantly increased in FLC. To test if this was a correlation or a causal relationship, the level of miRNA was increased by over-expression or decreased by expression of a miRZip miRNA. The results demonstrate that at least some of the changes in the miRNA in FLC may be responsible for the changes observed in the coding transcriptome.

## RESULTS

**Figure 1:**
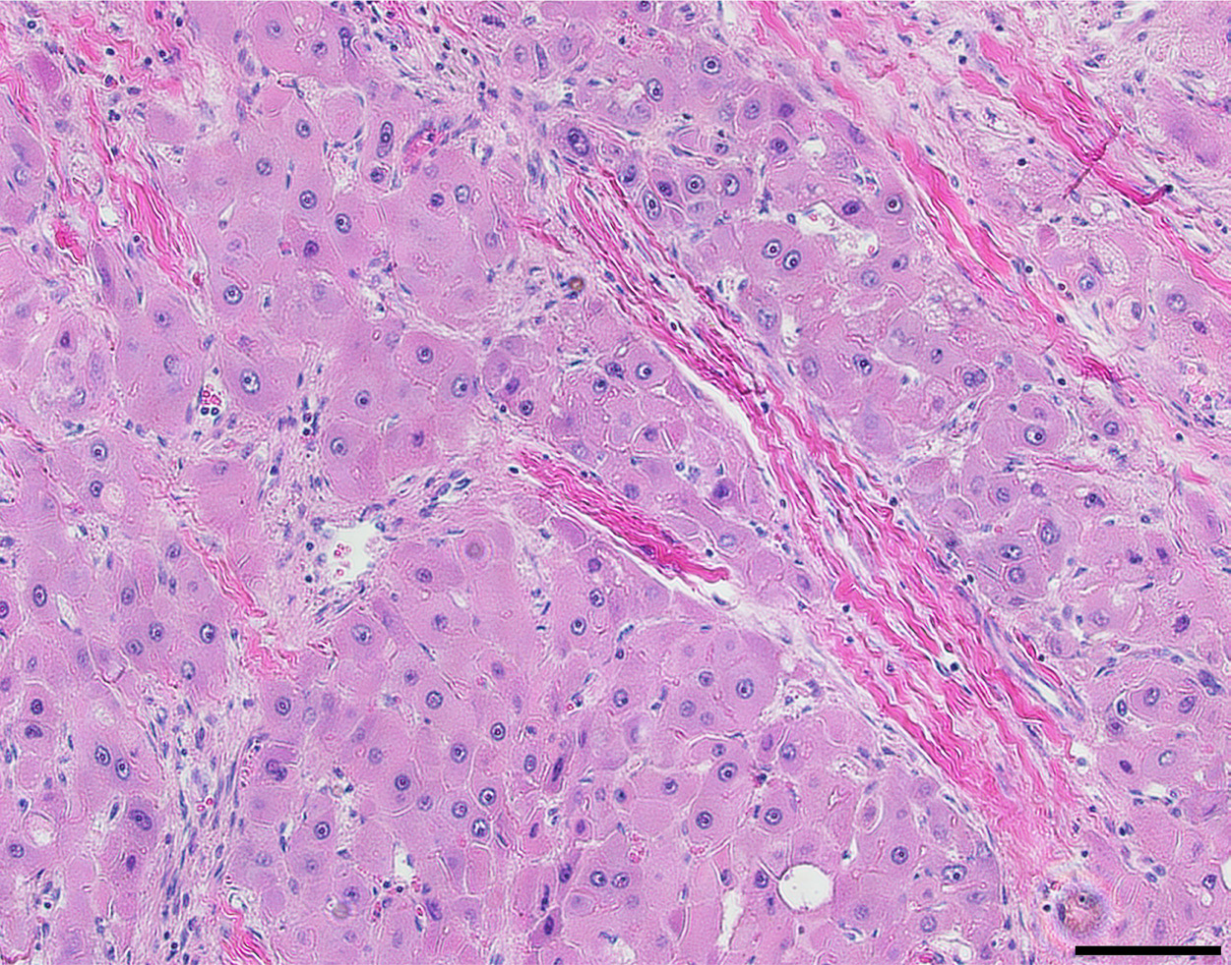
Hematoxylin and eosin stained FLC tumor tissue showing large polygonal cells with vesiculated nuclei, large nucleoli and intratumoral lamellar bands of collagen Scale bar = 100 μm.

**Figure 2:**
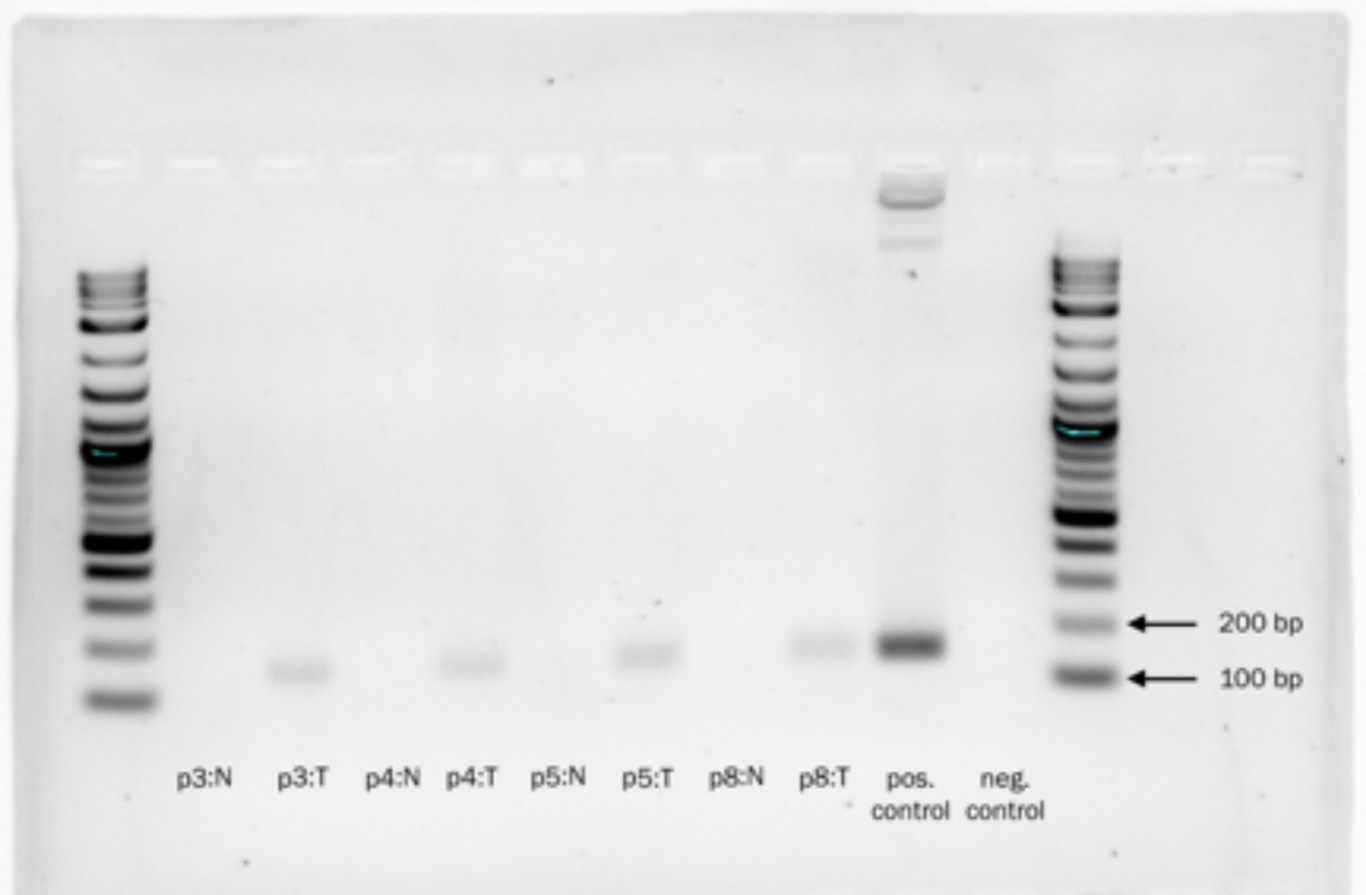
2% agarose gel demonstrating the presence of the *DNAJB1-PRKACA* chimeric transcript in tumor tissues (T = tumor), but not in adjacent normal liver samples (N = Normal) Log_2_ ladder. Expected amplicon 148 bp.

A pathologist experienced in the diagnosis of liver tumors (UKB) reviewed the histology from all FLC tumor samples. In all cases the FLC tumor tissue was distinguished by the presence of lamellar bands and large polygonal cells which were absent in the adjacent normal tissue on H&E (Figure 1). The tumor and adjacent normal liver tissues were tested for the presence of the *DNAJB1-PRKACA* chimeric transcript at the RNA level by PCR. The chimeric transcript was found in all of the tumor tissue samples, but not detected in the adjacent normal tissue (Figure 2).

**Figure 3:**
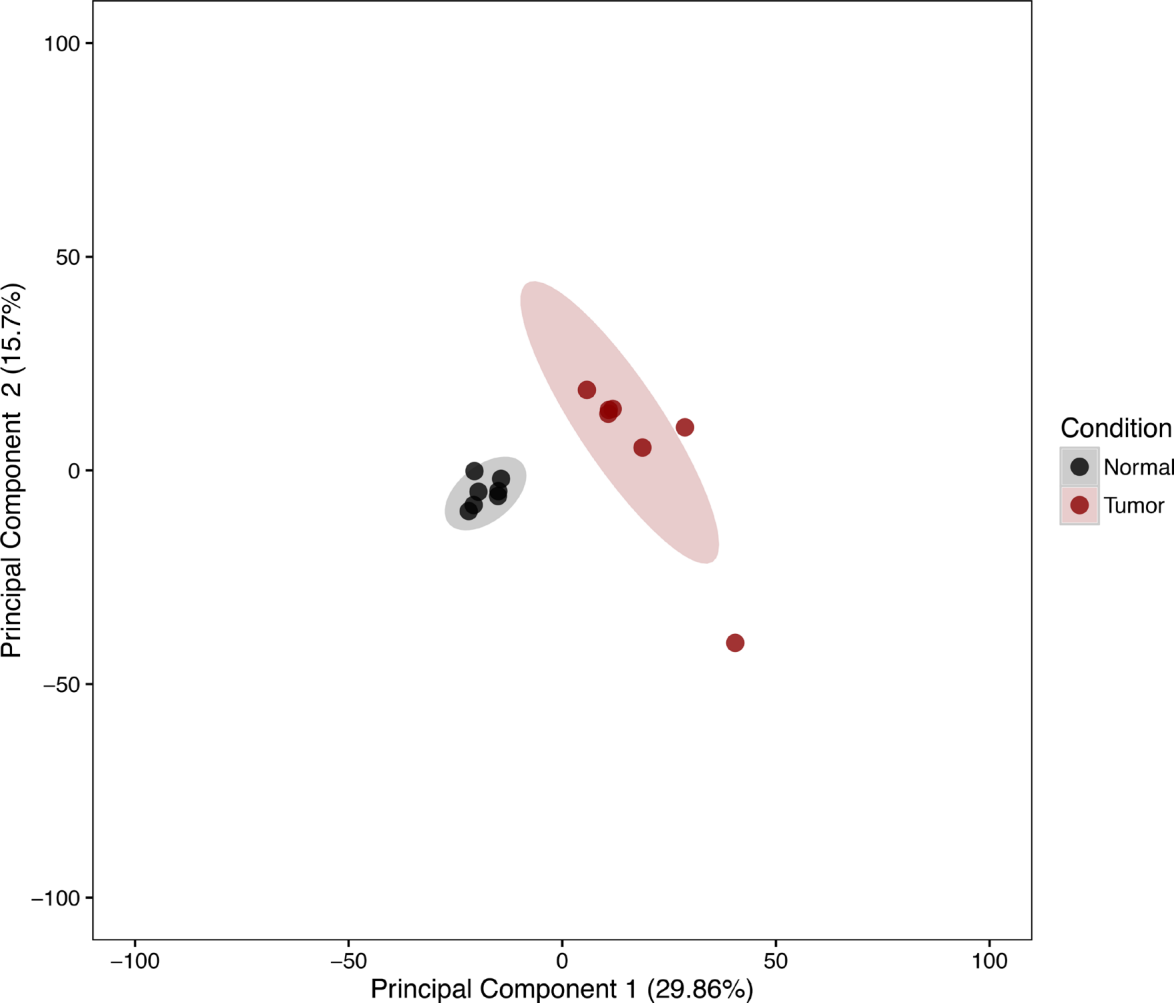
Principal component analysis of variance stabilized transformed miRNA RNA-Seq read counts of Tumor and Normal Samples Ellipses note 95% confidence interval. Axis percentages indicate variance contribution.

**Figure 4:**
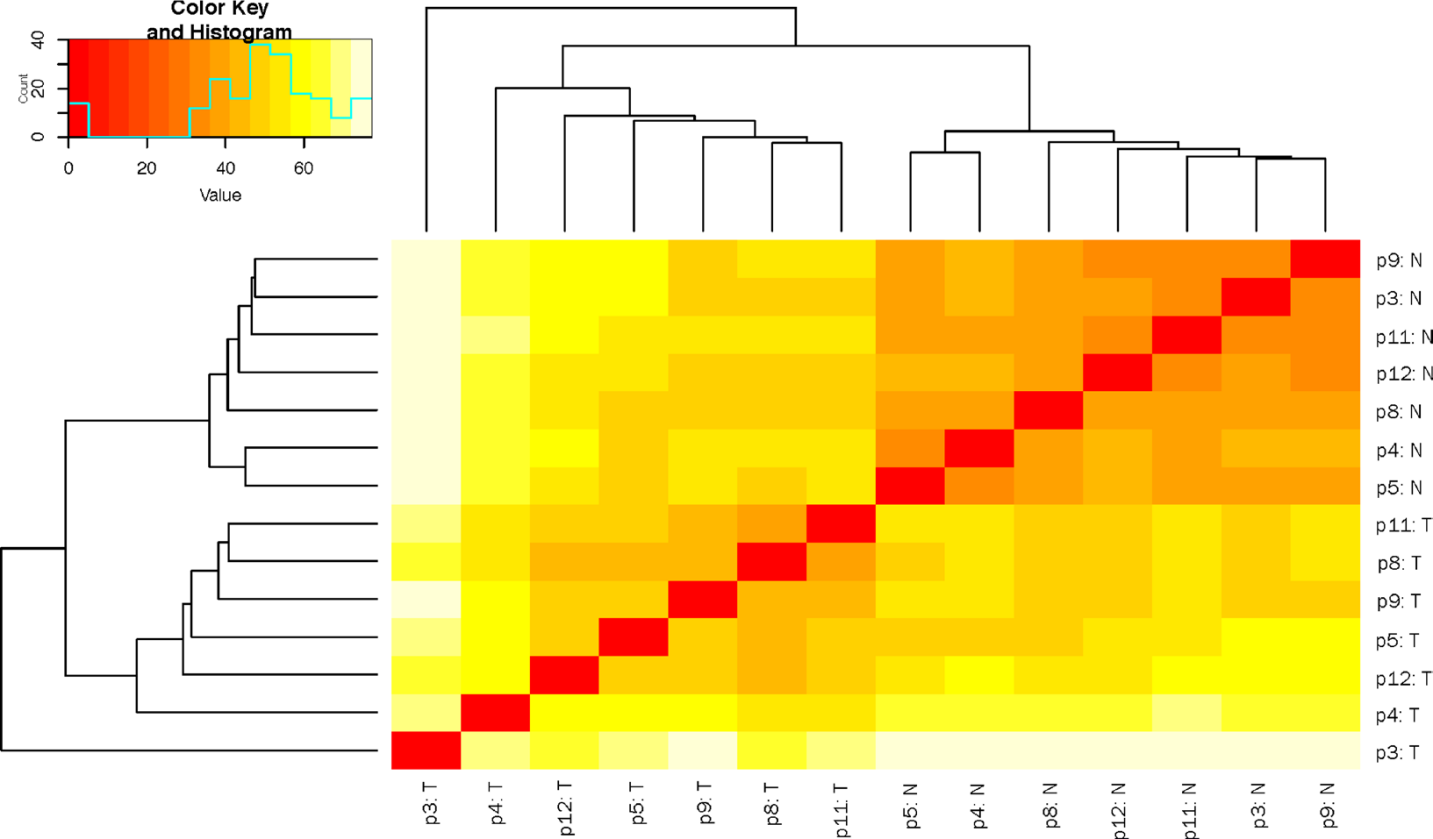
Heat map depicting hierarchical clustering of sample-to-sample distance of variance stabilized transformed small RNA-Seq read counts T = tumor, N = normal

**Figure 5:**
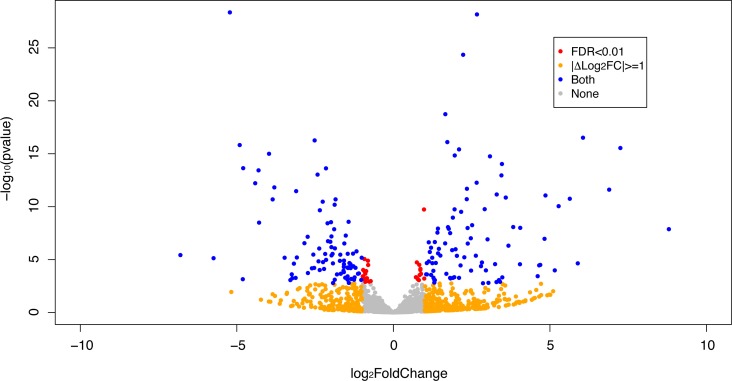
Volcano plot depicting differential expression results of miRNA in FLC tumor samples compared to paired adjacent normal liver Orange dots show |Log_2_ fold change |≥ 1, red dots show FDR ≤ 0.01 and blue dots correspond to miRNA that are both |Log_2_ fold change | ≥ 1 and FDR ≤ 0.01. Grey dots correspond to miRNA that do not meet any of the mentioned criteria.

**Figure 6:**
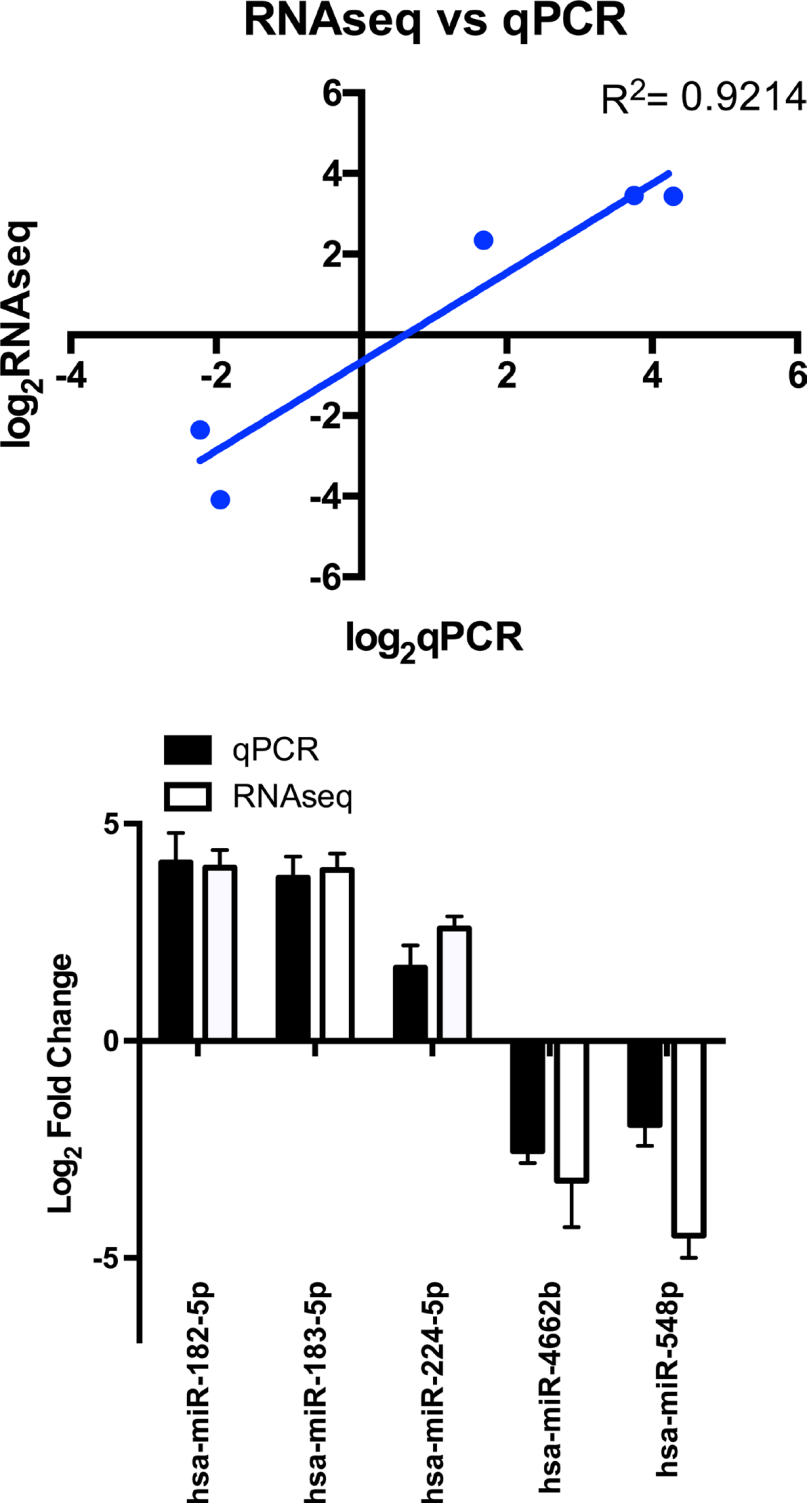
miRNA expression as assayed by RNAseq (*n* = 7) and qPCR (*n* = 5–8) qPCR data analyzed using the ΔΔC_T_ method referenced to SNORD42b. Bar graph shows Log_2_ fold change values yielded from RNAseq and qPCR for the same miRNA (R^2^ = 0.92), error bars indicate standard deviation.

The expression patterns of the miRNA, as quantified by RNA-Seq, in the FLC tissue were distinct from those seen in adjacent normal liver tissues and similar between tumor samples from different patients as evidenced by principal component analysis (Figure 3) and heat map unsupervised hierarchical distance clustering (Figure 4). Analysis from paired FLC tumor and adjacent normal liver samples (*n* = 7) showed 176 mature miRNA that were differentially expressed with an absolute log_2_ fold change ≥1 and a False Discovery Rate (FDR) ≤0.01 (Supplementary Table 1). Eighty-seven miRNA were under-expressed and 89 had increased expression in the FLC tumor samples as compared to paired adjacent normal liver samples (Figure 5). The differential expression observed in RNA-Seq was confirmed by qPCR for five miRNA. The log_2_ fold-change (tumor vs. normal) expression values as quantified by qPCR and RNA-Seq were similar (Figure 6, R^2^ = 0.92).

Differentially expressed mature miRNAs identified in the FLC tumor samples relative to adjacent normal liver were uploaded into DIANA miRPath microT-CDS v.3 [24] as separate data sets to identify their enrichment within various KEGG pathways. Using this method, the under-expressed mature miRNA showed enrichment of 49 KEGG pathways *p*-values at least <0.05, with correction for multiple testing using the FDR approach (Table 1). The over-expressed mature miRNAs yielded enrichment of 79 different KEGG pathways (Table 2). Numerous oncologically relevant KEGG pathways were highlighted between the two sets of analyses including Hippo, ErbB, and *wnt* signaling, among others. Many of these pathways, such as the EGF/ErbB2 and wnt pathways have already been shown to overexpressed in FLC in transcriptomic analysis.

**Table 1:** Top twenty-five KEGG pathways enriched with miRNA with Log_2_ fold change ≤-1 and FDR ≤ 0.01 in FLC tumors compared to normal liver as found in DIANA miRPath v3 Genes Union analysis. *P*-values corrected for multiple testing with FDR approach

KEGG pathway	*p*-value	#genes	#miRNAs
Mucin type O-Glycan biosynthesis	1.98E-16	26	35
Hippo signaling pathway	2.05E-07	117	74
Fatty acid biosynthesis	3.99E-07	10	24
TGF-beta signaling pathway	3.99E-07	65	65
Axon guidance	1.51E-06	96	69
Pathways in cancer	2.07E-06	288	81
Focal adhesion	6.78E-06	158	76
Renal cell carcinoma	1.69E-05	56	69
ErbB signaling pathway	1.69E-05	69	71
Proteoglycans in cancer	5.95E-05	144	74
Glutamatergic synapse	3.59E-04	85	67
N-Glycan biosynthesis	5.40E-04	36	56
Gap junction	5.40E-04	67	67
Adherens junction	5.40E-04	57	68
Endocytosis	8.08E-04	150	77
Colorectal cancer	1.05E-03	50	66
Thyroid hormone signaling pathway	1.61E-03	86	75
Rap1 signaling pathway	1.61E-03	149	79
Ras signaling pathway	1.98E-03	159	77
Ubiquitin mediated proteolysis	2.07E-03	100	69
Wnt signaling pathway	2.07E-03	102	72
Arrhythmogenic right ventricular cardiomyopathy (ARVC)	2.17E-03	51	60
Estrogen signaling pathway	2.26E-03	71	68
Regulation of actin cytoskeleton	2.66E-03	154	80
Morphine addiction	2.97E-03	67	75

**Table 2:** Top twenty-five KEGG pathways enriched with miRNA with Log2 fold change >1 and FDR < 0.01 in FLC tumors compared to normal liver as found in DIANA miRPath v3 Genes Union analysis

KEGG pathway	*p*-value	#genes	#miRNAs
Proteoglycans in cancer	5.30E-10	153	75
Bacterial invasion of epithelial cells	3.98E-07	68	66
Amphetamine addiction	7.48E-07	52	61
Pathways in cancer	7.48E-07	280	78
Glioma	1.57E-06	53	64
Hippo signaling pathway	1.78E-06	113	68
Renal cell carcinoma	2.55E-06	55	64
Prolactin signaling pathway	2.57E-06	58	62
ECM-receptor interaction	3.35E-06	56	62
Endocytosis	8.70E-06	150	74
ErbB signaling pathway	2.07E-05	70	73
Adherens junction	4.36E-05	59	68
Estrogen signaling pathway	4.97E-05	74	70
Morphine addiction	5.88E-05	69	64
Oxytocin signaling pathway	5.88E-05	119	70
Cocaine addiction	5.96E-05	37	59
N-Glycan biosynthesis	7.75E-05	37	51
Focal adhesion	1.57E-04	151	74
Pancreatic cancer	1.66E-04	52	63
Rap1 signaling pathway	1.66E-04	152	73
GABAergic synapse	2.55E-04	68	61
Thyroid hormone signaling pathway	2.80E-04	89	68
TGF-beta signaling pathway	3.27E-04	59	61
Glutamatergic synapse	3.28E-04	82	70

*P*-values corrected for multiple testing with FDR approach.

**Figure 7:**
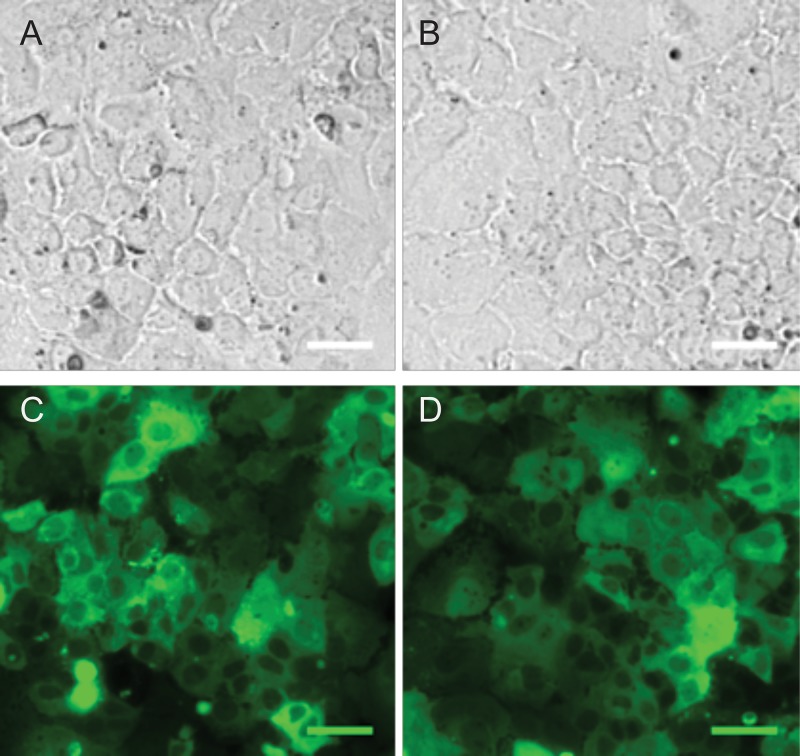
Verification of miRZip delivery Bright field 10× of Huh-7 cells transduced with lentivirus miRZip-548p (**A**) and lentivirus miRZip-scramble (**B**) and corresponding epifluorescent GFP images of the same field of cells (**C**, **D**). Scale bar = 50 μm

**Figure 8:**
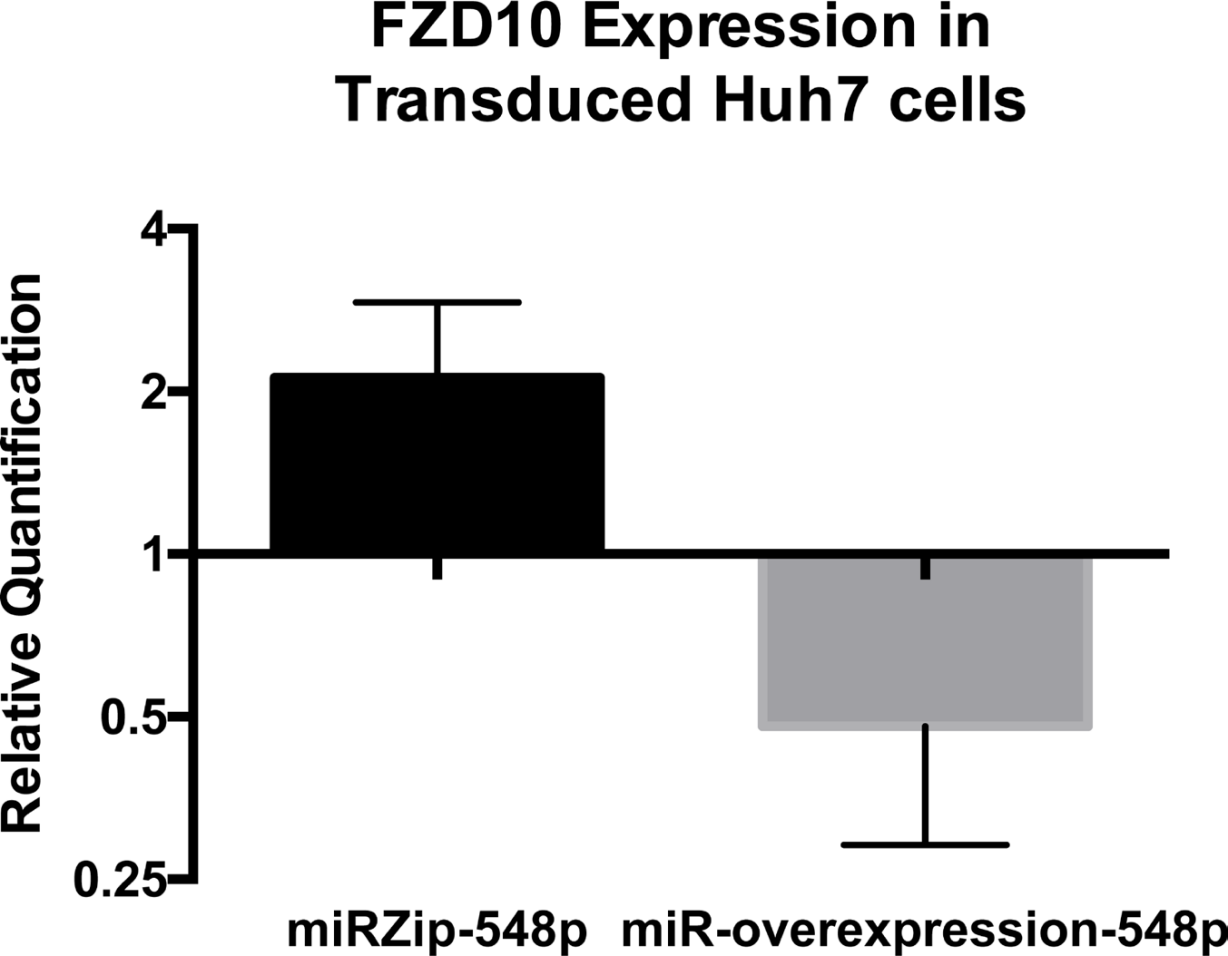
qPCR results of Frizzled 10 expression in Huh7 cells after transduction with miRZip-548p or miR548p over expression as compared to cells transduced with scramble sequence virus in biological triplicate (*p* = 0.026 and *p* = 0.024 respectively) miRZip-548p treated cells show over expression of FZD10, whereas over expression of miR-548p showed decrease in FZD10 in Huh7 cells. Analysis performed using the ΔΔC_T_ method as referenced to human β2-microglobulin. Statistical analysis performed with 2 tailed t-test. Error bars represent standard deviation.

**Figure 9:**
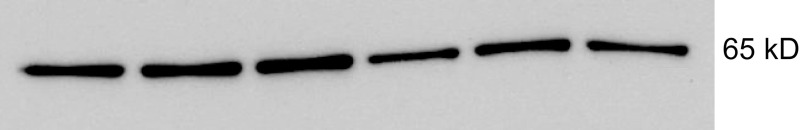
Immunoblot of FZD10 from lysates of Huh7 cells transduced with miRZip 548p (lanes 1–3) or scramble sequence (lanes 4–6) showing overexpression of FZD10 in cells transduced with miRZip 548p Expected molecular weight 65kD.

One miRNA, miR-548p, which was highly down-regulated in FLC (Δlog_2_ = −4.41, p_adj_ = 3.36 × 10^−11^), had target RNA transcripts predicted by DIANA that were previously described to be over-expressed in FLC tumor samples [7], in particular, the Frizzled 10 receptor (FZD10), a receptor of the *wnt* family. FZD10 mRNA is increased in FLC tumor relative to the adjacent normal tissue by Δlog_2_ = 6.13 with p_adj_ = 3.08 × 10^−20^ and the FZD10 protein is also increased in the FLC tumor (7). To examine whether there was a causal or corollary relationship between the changes in the non-coding RNA and changes in transcription, we tested if the relationship between the decreased expression of miR-548p and increased expression of FZD10. We increased expression of miR-548p via transduction with a pre-miRNA or decreased expression via transduction with an anti-miRNA (miRZip/MicroRNA Precursor Construct, System Biosciences, Palo Alto, CA) in the human heptatoma cell line, Huh7. The effects of over expressing the miRNA or miRZip on the expression of FZD10 were assayed by quantitative PCR and western blot (Figures 7–9). When miRZip was used to reduce miR-548p in Huh7 cells, we observed that the mRNA for FZD10 increased 2.12 times as referenced to beta 2 microglobulin (β2M) (*p* = 0.026, Figure 8 black bar). Likewise, the protein levels of FZD10 increased in comparison to Huh7 cells treated with scramble sequence (Figure 9). When Huh7 cells were transduced to increase expression of miR-548p, using a lentiviral microRNA precursor construct, we found the transcript of FZD10 was down regulated 0.48 times as referenced to β2M (*p* = 0.024) (Figures 7, 8, grey bar). Immunoblots of FZD10 in Huh7 cells treated with miRZip 548p show over expression of FZD10 in comparison to Huh7 cells treated with scramble sequence (Figure 9). The demonstration that decreasing miR-548p results in an increase of FZD10 and increasing miR-548p results in a decrease of FZD10, suggests a causal inverse relationship between this miRNA and the expression of this frizzled receptor in Huh7 cells.

We next focused our attention to the lncRNA to determine if they similarly show a statistically significant change between FLC and adjacent normal liver. Over 600 lncRNA significantly changed in expression ((|Δlog_2_|>1, p_adj_ < 0.01) (Supplementary Table 2 and a subset are given in Table 3). These changes in lncRNA, in general, did not correspond with those previously reported for other liver cancers, or any other cancers. Most of the most highly upregulated lncRNA have not been previously implicated in tumors and little is known of the functions of these lncRNA. In contrast, many lncRNA that are strongly increased in other liver cancers, did not increase in FLC. Some, such as HULC, were only slightly increased (Δlog_2_ = 1.45, p_adj_ = 1.5 × 10^−03^) in FLC, others such as HOTTAIR, were not increased (Δlog_2_ = 0.47, p_adj_ = not significant), and others, such as H19 were decreased (Δlog_2_ = −3.66 p_adj_ = 1.39 × 10^−9^).

**Table 3:** Differential expression of a subset of lncRNA which either have been implicated in other liver cancers or which show in FLC an absolute Log_2_ fold change ≥1 and FDR < 0.01 (complete table of lncRNA in FLC with significant changes is in Supplementary Table 2)

ENSEMBL (GRCh37/hg19)	ENSEMBL Name	GeneName	Log2FC	*p* value	*p* adjusted (FDR)
ENSG00000233179.1	Z82249.1	RP11-536P6.3	11.09	1.5E-21	4.8E-19
ENSG00000249111.1	AC108517.1	RP11-622J8.1	10.85	1.9E-21	5.9E-19
ENSG00000255327.1	AP003062.1	RP11-555G19.1	9.27	7.1E-22	2.3E-19
ENSG00000261182	AL596211.1	RP11-201A3.1	8.92	5.1E-67	2.1E-63
ENSG00000227674.1	LINC00355	LINC00355	8.45	3.1E-12	2.4E-10
ENSG00000249111	AC108517.1	RP11-622J8.1	7.13	3.4E-25	1.1E-22
ENSG00000215808	LINC01139	Link-A	6.83	7.9E-06	1.8E-04
ENSG00000235180	LINC00601	LINC00601	6.75	4.7E-27	1.7E-24
ENSG00000228430	AL162726.3	RP11-15B24.5	6.47	6.0E-22	1.3E-19
ENSG00000251292	ERVH-1	RP11-380P13.2	5.88	2.8E-20	4.9E-18
ENSG00000230234	AL162582.1	RP1-276N6.2	5.43	8.4E-14	5.3E-12
ENSG00000225006	AL929288.1	RP11-669M2.1	4.51	9.3E-09	2.0E-07
ENSG00000251164	HULC	HULC	1.46	2.3E-04	1.5E-03
ENSG00000228630	HOTAIR	HOTAIR	0.47	6.4E-01	NS
ENSG00000243766	HOTTIP	HOTTIP	0.20	8.4E-01	NS
ENSG00000226442	AC006037.1	AC006037.2	−1.39	1.7E-01	NS
ENSG00000247844	CCAT1	CCAT1	−1.84	2.5E-05	2.3E-04
ENSG00000237076	AL035706.1	RP5-836J3.1	−2.75	1.4E-04	1.1E-03
ENSG00000279218	AC006037.2	AC006037.2	−2.94	1.4E-04	1.0E-03
ENSG00000234223	AC003988.1	AC003988.1	−3.27	9.2E-07	1.2E-05
ENSG00000228980	LINC01205	RP11-702L6.4	−3.40	9.8E-07	1.3E-05
ENSG00000244128	LINC01322	RP11-85M11.2	−3.62	1.3E-07	2.1E-06
ENSG00000130600	H19	H19	−3.66	3.7E-11	1.3E-09
ENSG00000250266	LINC01612	RP11-789C1.1	−4.38	9.2E-10	2.4E-08
ENSG00000249173	LINC01093	RP11-701P16.4	−5.50	2.8E-14	1.9E-12
ENSG00000237949	LINC00844	LINC00844	−5.50	6.6E-10	1.8E-08

NS: Not significant.

## DISCUSSION

Liver cancer is the second most common cause of cancer death worldwide [25]. While advances in targeted treatment for advanced stage conventional HCC have been observed [26], no targeted therapies currently exist for FLC. Promising chemotherapeutic regimens have been reported in FLC patients [27], however, surgical resection remains the only consensus treatment with 5-year overall survival rates reported between 30–45% [28–30]. The DNAJB1-PRKACA chimera is the one structural variant common to FLC patients, making it a promising therapeutic target [8]. The transcriptomic analysis showing enrichment of numerous oncologically relevant pathways, points to additional new directions for therapies targeting pathways downstream of the drivers of the tumor [7]. In addition, the diagnosis of FLC remains problematic. Malouf and colleagues showed that inter-observer diagnostic reproducibility for the diagnosis of FLC, conventional HCC and cholangiocarcinoma between 12 pathologists was relatively poor [31]. This begs the question of developing molecular assays to aid FLC diagnosis as well as developing non-invasive screening and surveillance tests.

We have previously demonstrated that in primary FLC tumor tissue there are consistent changes in various oncologically relevant pathways such as EGF/ErbB2, Aurora Kinase A and *wnt* signaling pathways [7]. After examining the differentially expressed miRNA in FLC that meet statistical significance, we turned our attention to these same pathways that are enriched in the tumor to further explore the role miRNA may have in the pathogenesis of FLC. As seen in our KEGG pathway analysis (Tables 4, 5), many oncologically relevant pathways are highlighted within the top 10 results including: Hippo pathway, TGF-beta signaling, EGFR/ErbB signaling.

Investigations of the Hippo pathway have demonstrated activation of Yes-associated protein (YAP) yields increased hepatocyte proliferation. Alterations of the pathway are found during the process of liver regeneration, suggesting this pathway plays a role in modulating cell proliferation and liver size [32–34]. The Hippo signaling pathway has been implicated as a tumor suppressor pathway [33, 35] with inactivation leading to activity of the nuclear effector YAP. One group evaluated YAP activity in lung adenocarcinoma, colon adenocarcinoma and ovarian serous carcinoma, and found it to be commonly altered in the process of carcinogenesis [36]. A separate group further investigated YAP nuclear localization and staining pattern in pediatric hepatocellular carcinoma samples with a subset analysis of FLC vs non-FLC tumors. This study showed YAP nuclear localization in both HCC and FLC samples suggesting YAP activity, however YAP nuclear localization was found to a lesser degree in the FLC samples [37]. PKA, which is activated in FLC, phosphorylates YAP, which is an important downstream effector of the Hippo pathway [38]. In analyzing our data of under-expressed miRNA, 117 genes involved with Hippo signaling pathway are implicated as targets for 74 miRNA (*p* = 2.05 × 10^−7^). Similarly, for miRNA that are overexpressed, 113 genes involved with the pathway are implicated as targets for 68 miRNA (*p* = 1.78 × 10^−6^) suggesting its potential role in FLC.

Differentially expressed miRNA in FLC are also implicated to target *wnt* signaling. A role for *wnt* signaling in cancer was first described in the 1980s in the context of murine mammary cancer, but various gene mutations in this pathway have been identified in several cancers including: colorectal, breast, HCC and lung cancers among others [39–42]. In our previous analysis of the FLC transcriptome, we found a number of members of the *wnt* pathway demonstrated to be consistently overexpressed in FLC including: DKK4 (Δlog_2_ = 6.52, p_adj_ = 3.99 × 10^−19^), FZD10 (Δlog_2_ = 6.1, p_adj_ = 3.08×10^−20^), *wnt*16 (Δlog_2_ = 4.40, p_adj_ = 3×10^−03^) wnt1 (Δlog_2_ = 3.94, padj = 1.44 × 10^−1^), SFRP2 (Δlog_2_ = 3.25, padj = 3.84 × 10^−03^), LEF1 (Δlog_2_ = 2.55, p_adj_ = 7.57 × 10^−12^) [7]. With this knowledge, we directed our attention to transcripts in the *wnt* signaling pathway found to be highly over expressed, including FZD10, which has been implicated to have a role in colorectal cancer through activation of the β-catenin-TCF signaling pathway [43]. When looking at miRNA that were down-regulated in FLC, our DIANA miRPath v.3 analysis yielded enrichment of *wnt* signaling pathway, and more specifically, miRNA that were proposed to specifically target FZD10. The relationship between the decreased miR-548p and transcription of FZD10 in FLC is a correlation, which does not demonstrate causality. Further, for many mRNA there are numerous miRNA that limit their expression and eliminating one miRNA may not be sufficient to alter the level of transcription. To further investigate this relationship between miR-548p and FZD10 transcription level, we reduced the expression of miR-548p in Huh7 cells, which both increased the transcription of FZD10 and increase the protein level. Increasing the expression of miR-548p through viral transduction decreased the expression of FZD10 transcription. Given this observed relationship, miR-548p or FZD10 may guide future investigations looking into these as diagnostic or therapeutic targets for patients. This may indeed be a correlation seen *in vitro* as multiple miRNA may regulate one pathway; thus just because one miRNA is decreased does not necessitate that its target mRNA is increased, and further investigation is warranted.

MicroRNAs have been studied extensively and have proven to regulate multiple biological functions and disease states, including cancer [16, 44–46]. Overexpression, or silencing of miRNAs have been shown to play a particular role in disease progression and resistance or sensitivity to treatment [17, 46–55]. With our framework of identifying the most aberrantly expressed miRNA in FLC, we provide a scaffold for future studies using miRNA in FLC.

When viewing these miRNA that are differentially expressed with statistical significance in conventional HCC and comparing them to those found in FLC, we see that many of them are not represented in our dataset, such as miR-210, miR-373, and miR136, among others [56]. Wang *et al*. previously investigated miRNA expression in conventional HCC and found miR-224 to be one of the most highly over expressed miRNA in HCC tissue in comparison to normal liver [57]. While miR-224 was found to be over expressed in our dataset, it was not the most highly over expressed miRNA and it did not appear to be as highly over expressed as in HCC as reported by Wang. Among other previously described down regulated miRNAs in conventional HCC is miR-122, accounting for near 70% of the miRNA population in the adult liver acting as a key regulator of cholesterol and fatty-acid metabolism [56, 58–60]. While the down-regulation of miR-122 has been detected in more than 70% of HCC, it was not among the most highly down regulated observed in our dataset.

Several investigations have examined the noncoding lncRNA and miRNA landscape of conventional HCC in the context of the native tumor tissues and as circulating biomarkers of disease. The profile of lncRNA in FLC was very different from HCC. Some lncRNA that increased significantly in conventional HCC, were only slightly increased in FLC. We expected the highly upregulated in liver cancer (HULC) to be upregulated in FLC. HULC, first shown to be very upregulated in HCC over ten years ago [20] upregulated in cholangiocarcinoma [61], and is upregulated by PKA phosphorylation of CREB [62], as well as in many cancers leading to the proposal that it is a prognostic biomarkers for human cancers [63]. Surprisingly, in FLC, HULC is only increased slightly (Δlog_2_ = 1.45, p_adj_ = 1.5 × 10^−03^). Expression of the lncRNA HOTAIR, which has been shown to correlate with tumor recurrence in HCC [64–66], and supports tumor invasion HCC [67] was not found to be increased in FLC (HOTAIR Δlog_2_ = 0.47, p_adj_ = not significant). Others that are increased in HCC and cholangiocarcinoma, such as H19 which drives cell growth and invasion [68], was found to be decreased in FLC (Δlog_2_ = −3.66 p_adj_ = 1.39 × 10^−9^).

There were near 300 lncRNA that were increased, and over 300 that were decreased significantly in expression in FLC (|Δlog2|>1, p_adj_ < 0.01) (Table 6). Even as this manuscript was in progress, the annotation of lncRNA was constantly changing making it difficult to assign a precise number of “significantly altered” transcripts [69, 70]. Notably, a few of these lncRNAs significantly altered have been previously implicated in cancers. One example is the long intergenic non-coding RNA for kinase activation, Link-A (linc01139, (Δlog_2_ = 6.83, p_adj_ = 1.8 × 10^−4^), is highly expressed in breast cancer and has been shown to specifically interact with phosphatidylinositol 3,4,5-trisphosphate (PIP3) and Protein Kinase B (PKB/AKT) [71]. The interactions of Link-A are important for recruitment and activation of AKT as well as resistance to inhibitors of AKT. A second example, found to be highly overexpressed is linc00355 (Δlog_2_ = 8.45, p_adj_ = 2.4 × 10^−10^), which has previously been observed to be overexpressed in bladder cancer. However, in a study by Seitz and colleagues evaluating lncRNA candidate oncogenes, a reduction in expression of linc00355 had no effect on cell viability [72]. Many of the other lncRNA that are highly altered in expression have not been previously correlated with tumors. For most of these little is known of their normal physiology nor their interactions. A few have been implicated to interact with miRNA. RP11-622J8.1 has been implicated in interacting with the miRNA: hsa-miR-16-1-3p, hsa-miR-4698, hsa-miR-27a-3p, hsa-miR-27b-3p, hsa-miR-3662, hsa-miR-5571-5p, hsa-miR-570-3p, hsa-miR-3658, hsa-miR-519e-3p, hsa-miR-514b-5p and hsa-miR-515-3p [69, 70]. However, not enough is known of the physiological implications of these interactions to determine if they are only correlations with FLC or if there is a causal link. While this paper was in preparation it was reported that four of the lncRNA seen in Supplementary Table 2 were observed increased in FLC on a search of TCGA data, which might include our data set [73]: AF064858.6 (also known as lnc-ERG-9:5 Δlog_2_ = 4.92, p_adj_ = 1.02 x10^−9^); linc0313(Δlog_2_ = 3.27, p_adj_ = 3.3×10^−5^), linc00473(Δlog_2_ = 5.86, p_adj_ = 7.9×10^−13^) and RP11-157N3.1 (also known as linc01307, Δlog_2_ = 3.38, p_adj_ = 9.9×10^−3^). To evaluate which of these are causally related to tumorigenesis will require an analysis similar to the work presented here on miR-548p.

Prior investigations have shown circulating miRNA are relatively resistant to native RNase activity in the blood, making them potential non-invasive biomarkers of disease for various cancers [13, 74–76]. Yamamoto *et al.* previously reported serum elevations of miR-500 in HCC patients with levels that subsequently declined after surgical treatment in a small group of patients [75]. Similarly, Shigoka and colleagues found decreased miR-92a in serum samples of patients with HCC compared to healthy counterparts, with elevation after surgical treatment [77]. MicroRNA are clearly differentially expressed between FLC tumor and the normal liver. While the functional interactions of many of these miRNA are still unknown, the clear differential expression of these miRNA may still serve as a foundation for further research aimed at developing non-invasive biomarkers of FLC.

Our results suggest a unique expression profile for non-coding RNA (lncRNA and miRNA) in FLC. Our data bears little similarity to the non-coding RNA expression in conventional HCC or cholangiocarcinoma, which is again consistent with our previous characterization of the coding RNA. Furthermore, FLC behaves as a different disease from other liver pathologies as evidenced by the tumor biology and epidemiology. Due to its advanced stage at diagnosis and poor prognosis, this disease still desperately requires further investigation for novel treatments and diagnostic tools.

## MATERIALS AND METHODS

### Human tissue samples

With Institutional Review Board approval (Rockefeller IRB# SSI-0797, SSI-0798 and Memorial Sloan Kettering Cancer Center IRB Protocol #13-010), we obtained samples of OCT embedded tumor and adjacent normal liver tissue. Diagnosis of FLC was confirmed for all tumor samples by a single pathologist (UKB) specializing in hepatobiliary tumors. All tumor samples were confirmed to have >80% tumor cells with minimal necrosis, which was macro-dissected away with stromal components. Review of adjacent normal liver samples revealed no evidence of tumor infiltration.

### RNA isolation, generation of cDNA and sequencing

Total RNA was extracted from cells in culture, or from optimal cutting temperature (OCT) embedded frozen FLC tumor and adjacent normal liver tissue samples using the miRNeasy Mini Kit (Qiagen). The miScript II RT Kit with was used to convert total RNA into cDNA as described by the manufacturer, with use of the HiSpec buffer when used for miRNA and HiFlex buffer when used for mRNA (Qiagen). RNA-Seq libraries were generated using the TruSeq small RNA sample preparation kit (Illumina, San Diego, CA) following the manufacturer's protocol. Libraries were sequenced with 1 × 50 bp single-end reads on an Illumina HiSeq 2000 system. Small RNA reads were mapped and quantified with miraligner and quantified read counts were annotated with Gencode v19. Differential miRNA expression analysis was performed using R version 3.2.3 [78] with the Bioconductor package DESeq2 [79] incorporating variable factors for condition of tumor or normal and patient. For the lncRNA, RNA-Seq libraries were prepared using TruSeq Stranded Total RNA Sample Prep Kit with Ribo-Zero ribosomal RNA depletion (Illumina). The manufacturer's protocols were used to sequence ribosomal RNA-depleted libraries at 2 × 50-bp paired-end reads on an Illumina HiSEq. 2500 in high-output mode, to an average depth of 86 × 10^6^ paired-end reads per sample (range 43 × 10^6^ to 139 × 10^6^).

### Verification of the DNAJB1-PRKACA chimeric transcript

cDNA of tumor tissues was prepared as described above. PCR amplification of the *DNAJB1- PRKACA* chimera was conducted using 45 μL Platinum PCR mix (Invitrogen), 2 μL each of 5 μM forward and reverse primers (Supplementary Table 3), and 1 μL cDNA template for total reaction volume of 50 μL. Primers for the chimera were designed with MacVector's Primer Design (Primer3) (version 13.5.5) and purchased from Integrated DNA Technologies. Cycling conditions were initial activation of 95°C for 15 min followed by 40 cycles of 94°C for 15 seconds, 55°C for 30 seconds and 70°C for 30 seconds. PCR products were loaded into a 2% agarose gel, pre-stained with SYBR safe (Invitrogen) was run at 100V for 45 minutes and then imaged with a BioRad Gel Doc EZ imager.

### Real time PCR

Real Time PCR was performed in triplicate on an Applied Biosystems QuantStudio 12K Flex instrument console using QuantiTect SYBR green based PCR master mix (Qiagen) according to the manufacturer's instructions with the modification of total reaction volume being 25 μL. Cycling conditions were: initial activation step 95°C for 15 min followed by 40 cycles of 94°C for 15 sec, 55°C for 30 sec and 70°C for 30 sec. QuantiTect and miScript primers were purchased from Qiagen. Levels of RNA expression were determined using the QuantStudio 12K Flex Software version 1.2.2 (Applied Biosystems), and Microsoft Excel. Data were analyzed by the ΔΔC_T_ method after normalization to human beta 2 microglobulin (B2M), or small nucleolar RNA (SNORD42b) where appropriate. 2-tailed *t*-tests were used to identify statistical significance with *p* < 0.05 as threshold for significant values.

### miRNA pathway analysis

Mature miRNA targets from DESeq2 analysis were filtered for absolute log_2_ fold change ≥1 with a false discovery rate (FDR) ≤ 0.01. Selected miRNA were placed in DIANA-miRPath version 3 microT-CDS to analyze pathways enriched with gene targets of miRNA [24].

### Cell culture

HEK293T and Huh7 cell lines were maintained in Dulbecco's Modified Eagle's medium (DMEM, Life Technologies) supplemented with 10% heat-inactivated fetal bovine serum (Sigma) and 1% non-essential amino acids (Gibco). Adherent cells were grown in an incubator at 37°C with 5% CO_2_ (Fisher Scientific).

### Generation of Lentivirus and miRNA overexpression/knock down

HEK 293T cells were seeded in a 10 cm plate and allowed to reach 60% confluence in DMEM supplemented with 10%FBS and 1%NEAA. Prior to transfection, media was replaced with fresh DMEM media supplemented with 3% FBS and 1%NEAA. Cells were co-transfected with 5 μg of pCRV1-NL-gag/pol, 1 μg of VSV-G and 5 μg of appropriate miR-Zip or miRNA precursor overexpression plasmid (System Biosciences, Mountain View, CA) along with 44 μL of polyethylenimine (PEI) and OptiMem (Gibco). Cells were incubated at 37°C for 48 hr and virus was harvested and filtered through a 0.45 μm filter. Polybrene 4 μg/mL (Milipore) and HEPES 20 mM (Gibco) were added to viral stocks and was preserved at −80°C until used. Huh7 cells were transduced with 1 mL of the appropriate virus. After 48 hours, cells were imaged to detect GFP signal, and 2 μg/mL of puromycin was added to the cell media of miR-Zip transduced cells for lentiviral selection.

### Imaging

Huh-7 cells transduced with lentiviral constructs in 6 well tissue culture dishes were imaged 48 hours after transduction using an Olympus IX70 microscope with a 10× UPlanFL N air objective (N.A. 0.3), and an Olympus xenon arc-lamp. GFP images were obtained using a Semrock excitation band pass filter (FF01-466/40-25-D) with a Di02-R488-26×38-EP beam splitter, and an emission band pass filter (FF01-525/30-25-D). The image acquisition and data analysis were done using MetaMorph software version 7.7.8.0 (Molecular Devices, LLC, Sunnyvale, CA). Images were captured with an ORCA-ER C4742-95-12ER Hamamatsu camera using an exposure time of 500 ms for bright field images and 50 ms for fluorescent images. H&E slides of FLC were imaged on an Olympus IX83 microscope using a 10× objective. H&E color images were captured using an Olympus DP26 camera.

### Immunoblotting

Huh7 cells previously transduced with lentivirus were detached from culture dishes using ice-cold 50 mM EDTA/PBS and a cell scraper (Corning, Corning, NY). Cells were centrifuged at 2000 RPM for 5 minutes at 4°C, and the supernatant aspirated. Cell pellets were lysed in RIPA buffer (Sigma) containing protease and phosphatase inhibitors (Complete EDTA-free and Phosphostop, Roche, Indianapolis, IN) on ice. Protein concentrations were measured by a modified Lowry assay (DC protein assay, Bio-Rad, Berkeley, CA). 10μg of protein per sample were diluted with 4X Nupage LDS sample buffer (Life technologies) containing 10% β-mercaptoethanol. Samples were heated at 100°C for 5 minutes, and then loaded on 4–12% Bis-Tris gels (Nupage, Invitrogen, Carlsbad, CA) and run in MOPS buffer for 50 minutes at 200V. Transfer was performed using the iBlot (Life Technologies, Carlsbad, CA). Membranes were blocked for 1 hour in 5% milk in TBST, washed in TBST, and then probed with primary antibodies in 5% milk against either human FZD10 (Proteintech, 18175-1-AP, 1:500); or human β-Actin (Sigma, A5316, 1:1000); and incubated overnight shaking at 4°C. After washing in TBST, membranes were incubated with horseradish peroxidase-conjugated appropriate secondary antibodies (Sigma, A0545 goat anti rabbit, A9917, goat anti mouse, 1:100,000) in 5% milk in TBST for 1 hour. Membranes were washed in TBST and then incubated with Amersham ECL prime western blotting detection reagent (GE Healthcare).

## SUPPLEMENTARY MATERIALS TABLES






