# Supplementary material for: Non coding RNA analysis in fibrolamellar hepatocellular carcinoma
Source: Oncotarget. 2017 Dec 15;9(12):10211–27. doi: 10.18632/oncotarget.23325 (PMC5828204; doi:10.18632/oncotarget.23325)
Supplement: Supplementary file 1 [file oncotarget-09-10211-s001.pdf]

Non coding RNA analysis in fibrolamellar hepatocellular carcinoma

SUPPLEMENTARY MATERIALS

Supplementary Table 1: Differential expression of mature miRNA with absolute Log_2 fold change ≥1 and FDR<0.01. See Supplementary_Table_1

Supplementary Table 2: Differential expression of lncRNA with absolute Log_2 fold change ≥1 and FDR<0.01. See Supplementary_Table_2

Supplementary Table 3: Chimera primer sequences

Primer	Sequence 5′→3′
Chimera Forward	TTCAAGGAGATCGCTGAGGC
Chimera Reverse	CTGTGTTCTGAGCGGGACTT

Expected amplicon 148 bp
